# Involution perception and problematic smartphone use among Chinese college students: the indirect role of anxiety and the pathway-specific moderating role of psychological resilience

**DOI:** 10.3389/fpsyg.2026.1914988

**Published:** 2026-08-21

**Authors:** Chong Yang, Jingyi Hu

**Affiliations:** 1 Psychological Counseling Center, Guangdong University of Finance and Economics, Guangzhou, China; 2 Psychology Teaching and Research Office, Guangzhou Vocational College of Technology and Business, Guangzhou, China

**Keywords:** anxiety, college students, involution perception, moderated mediation, problematic smartphone use, psychological resilience

## Abstract

**Background:**

Involution, or “Neijuan” in Chinese, has recently been operationalized as escalating and often irrational competition characterized by increased effort with diminishing returns. Although involution has become increasingly salient in Chinese higher education, little is known about its association with problematic smartphone use (PSU) among college students. Grounded in the Interaction of Person-Affect-Cognition-Execution (I-PACE) model and Conservation of Resources (COR) theory, this study examined whether anxiety mediated the association between involution perception and PSU, and whether psychological resilience moderated this indirect association. Given that resilience is typically viewed as a protective factor but may also facilitate sustained, instrumental engagement under pressure, this study explored its potential pathway-specific moderating roles.

**Methods:**

A cross-sectional survey was conducted among 1,002 undergraduate students from a university in Guangzhou, China (573 female, 429 male; mean age = 20.10 years, SD = 1.35). Participants completed validated self-report measures assessing involution perception (encompassing psychological pressure, social norms, competitive behaviors, and resource scarcity), PSU, anxiety, and psychological resilience. A moderated mediation model (PROCESS Model 8) was tested with 5,000 bootstrap samples, controlling for age and gender.

**Results:**

Involution perception was positively associated with PSU and anxiety, while psychological resilience was negatively associated with all three variables. Anxiety significantly mediated the association between involution perception and PSU. Psychological resilience moderated both pathways, but in opposite directions. As hypothesized, the positive association between involution perception and anxiety was weaker at higher levels of resilience. However, the direct association between involution perception and PSU was significantly stronger at higher levels of resilience.

**Conclusion:**

The findings reveal a pathway-specific role of psychological resilience in the context of involution. While resilience buffers the emotional toll of competitive pressure by reducing anxiety, it simultaneously amplifies the direct behavioral reliance on smartphones, potentially reflecting sustained, high-intensity engagement in a demanding environment. This pathway-specific effect suggests that resilience is not a uniformly protective factor. The results underscore the utility of targeting anxiety to mitigate PSU and highlight the need for nuanced interventions that distinguish between reducing emotional distress and managing device-dependent coping patterns in competitive educational settings.

## 1 Introduction

The term “involution” (Neijuan in Chinese) has recently been operationalized in Chinese psychological research (Liu et al., 2022; Zhang et al., 2024), referring to escalating, often irrational competition with diminishing returns. In university settings, this phenomenon is particularly salient because academic evaluation, peer comparison (see also Chou and Edge, 2012; Vogel et al., 2014), career uncertainty, and pressure for self-optimization often converge during emerging adulthood. In university settings, involution is particularly salient because academic evaluation, peer comparison, career uncertainty, and pressure for self-optimization often converge during emerging adulthood. Students who perceive that they must invest ever-increasing effort merely to maintain their relative standing may experience heightened strain and become more likely to rely on maladaptive coping strategies. Recent evidence has suggested that an academic involution atmosphere is associated with mental exhaustion among college students (Liu et al., 2024). Importantly, involution perception is a multidimensional construct that captures not only the subjective experience of psychological pressure but also perceived social norms that prescribe relentless effort, overt competitive behaviors among peers, and a sense of resource scarcity in which opportunities appear increasingly limited (Zhang et al., 2024). Unpacking how these dimensions collectively relate to student wellbeing and behavior represents an important line of inquiry.

One behavioral outcome that may be particularly relevant in this context is problematic smartphone use (PSU). PSU refers to excessive, poorly controlled, or maladaptive smartphone use that is accompanied by functional impairment in daily life (Billieux et al., 2015; Kwon et al., 2013). Among college students, smartphones are deeply embedded in studying, communication, entertainment, and social interaction. Although smartphones provide convenience and support for everyday functioning, persistent overreliance may interfere with concentration, academic performance, sleep (Carter et al., 2016), and psychological wellbeing. Prior research has linked PSU to a range of emotional and psychosocial difficulties, particularly anxiety, stress, and deficits in adaptive coping (Elhai, 2019; Sohn et al., 2019; Yang et al., 2021; Samaha and Hawi, 2016). More recent meta-analytic evidence has confirmed the robust association between academic burnout and problematic smartphone use (Wang et al., 2026). Academic burnout is a construct conceptually related to perceived involution. Cross-cultural studies have further highlighted the mediating roles of belongingness and fear of missing out in the relationship between emotional distress and smartphone addiction (Akinci and Durmus, 2025). Broad reviews have further documented the adverse effects of social media and digital technologies on adolescent well-being (Kross et al., 2021; Odgers and Jensen, 2020). However, relatively little is known about whether students’ perception of involution in the academic environment is associated with PSU. This perception is characterized by felt pressure, internalized norms of overwork, competitive vigilance, and zero-sum thinking. Examining this association may help extend understanding of whether culturally and contextually embedded competitive pressures are associated with maladaptive technology-related behaviors in university populations.

The present study is informed by the Interaction of Person-Affect-Cognition-Execution (I-PACE) model (Brand et al., 2016, 2019), which proposes that problematic technology-related behaviors develop through interactions among distal person or contextual variables, affective and cognitive responses, and behavioral regulation processes. Within this framework, involution perception may be conceptualized as a distal contextual factor that contributes to emotional strain and shapes the likelihood of maladaptive smartphone use. When students perceive pervasive and inescapable competition, they may experience greater psychological burden and may become more likely to turn to smartphones for distraction, reassurance, or temporary relief. These pressures are amplified by norms glorifying overwork and a sense of shrinking opportunities. From this perspective, involution perception may be meaningfully associated with PSU through affective responses that arise in stressful competitive environments.

The Conservation of Resources (COR) theory (Hobfoll, 1989) provides an additional framework for understanding this association. COR theory proposes that psychological stress emerges when valued resources are threatened, lost, or insufficiently replenished. In the context of college life, involution perception may reflect a sustained sense of resource threat, as students feel pressured to devote increasing amounts of time, energy, and attention without commensurate returns. The perceived scarcity of opportunities and the relentless competitive behaviors modeled by peers may further heighten this sense of resource depletion. Under such circumstances, smartphone use may become an easily accessible coping response. Although this response may provide temporary relief, it may also contribute to maladaptive or excessive use patterns over time. Taken together, the I-PACE model and COR theory suggest that involution perception may be associated with PSU both directly and indirectly through emotional processes.

Anxiety may be a particularly important affective correlate in this process. Students who perceive intense and unavoidable competition may become more concerned about academic performance, future opportunities, and social evaluation. This heightened anxiety may, in turn, be associated with greater reliance on smartphones as a means of distraction, emotional regulation, reassurance seeking, or escape from stress. Previous studies have consistently emphasized the importance of emotional vulnerability and distress-related pathways in PSU (Canale et al., 2021; Elhai, 2019; Elhai et al., 2021; Tandoc et al., 2015). Moreover, anxiety has been repeatedly identified as a factor associated with greater engagement in problematic digital behaviors among adolescents and young adults. Accordingly, it is plausible that involution perception is positively associated with anxiety and that anxiety is, in turn, positively associated with PSU. On this basis, anxiety may statistically account for part of the association between involution perception and PSU.

Psychological resilience may further shape this process. Resilience is generally defined as the capacity to adapt effectively in the face of adversity, stress, or change (Connor and Davidson, 2003; Southwick et al., 2014). Within the COR framework, resilience can be viewed as a personal resource that helps individuals cope with stress, maintain psychological functioning, and reduce emotional depletion. Students with higher resilience may therefore show a weaker association between perceived involution and heightened anxiety. In this sense, resilience may buffer the emotional consequences of competitive stress, consistent with its traditionally protective role.

At the same time, resilience may also be relevant to the direct association between involution perception and PSU, although its effects may not be uniformly protective across all pathways. Two competing perspectives can be delineated. On the one hand, if resilience operates as a global protective factor, it would be expected to attenuate the direct link between involution perception and PSU; highly resilient students would be less likely to resort to excessive smartphone use even under intense competitive pressure. On the other hand, an emerging body of work suggests that resilience can, in certain demanding contexts, facilitate sustained goal-directed engagement and persistence, which may paradoxically be associated with greater behavioral dependence on productivity tools (Britt et al., 2016; Hao et al., 2023). In highly competitive academic environments, students with higher resilience may remain more actively immersed in academic demands, social coordination, and information management, thus using smartphones more intensively as tools for performance maintenance. Under such conditions, smartphone use may represent not purely dysregulated escape but a high-intensity coping style that blurs the boundary between functional and problematic use. Examining resilience as a moderator may therefore provide a more nuanced understanding of how students respond to involution-related pressure, and this study specifically explored whether resilience might function in a pathway-specific manner.

Based on this reasoning, the present study aimed to examine the association between involution perception and PSU among Chinese college students and to test a moderated mediation model in which anxiety served as a mediator and psychological resilience moderated both the association between involution perception and anxiety and the direct association between involution perception and PSU. By focusing on a culturally salient form of perceived competitive pressure, this study seeks to extend the literature on PSU beyond general stress-based accounts and to contribute to a more context-sensitive understanding of maladaptive smartphone use in higher education.

Accordingly, four hypotheses were proposed:

*H1*: Involution perception would be positively associated with PSU.

*H2*: Anxiety would mediate the association between involution perception and PSU.

*H3*: Psychological resilience would moderate the association between involution perception and anxiety, such that the positive association would be weaker at higher levels of resilience.

*H4*: Psychological resilience would moderate the direct association between involution perception and PSU. Given the competing theoretical possibilities outlined above, no directional hypothesis was specified for this moderation. Instead, this study explored whether resilience would attenuate (consistent with a global protective model) or amplify (consistent with a sustained engagement model) this direct pathway.

## 2 Methods

### 2.1 Participants and procedure

This study employed a cross-sectional design with convenience sampling. Participants were undergraduate students recruited from Guangdong University of Finance and Economics in Guangzhou, Guangdong Province, China. Students from the first through fourth undergraduate years were invited to complete an online questionnaire survey. Prior to participation, all participants provided electronic informed consent after reading a detailed information sheet that outlined the study’s purpose, procedures, potential risks and benefits, confidentiality assurances, data handling procedures, and their right to withdraw at any time without penalty. All participants were aged 18 years or older and were competent to provide consent on their own behalf.

A total of 1,226 questionnaires were initially collected. Following the examination of response patterns, cases were excluded based on three criteria: (a) patterned answering (e.g., straight-lining, diagonal responding; 98 cases excluded), (b) excessively short completion times (less than 180 s; 76 cases excluded), and (c) substantial missing data (more than 20% of items unanswered; 50 cases excluded). These criteria were applied sequentially, resulting in the exclusion of 224 cases in total. After data screening, 1,002 valid cases were retained for the final analyses, yielding an effective response rate of 81.73%. For the retained cases, a small proportion of items had missing responses (less than 20% per case). Missing values were handled using series mean imputation within each scale, a conservative approach that preserves sample size while minimizing distortion of scale-level distributions. The final sample included 573 female students (57.2%) and 429 male students (42.8%), with a mean age of 20.10 years (SD = 1.35, range: 18–24 years). Regarding academic year, 268 (26.7%) were first-year students, 341 (34.0%) were second-year students, 274 (27.3%) were third-year students, and 119 (11.9%) were fourth-year students. We acknowledge that second-year students constituted a relatively larger proportion of the sample than other year groups. This overrepresentation reflects differential participation rates rather than the university’s actual enrollment distribution. During the survey period, first-year students had particularly intensive course schedules, which limited their available time for non-mandatory surveys. Third- and fourth-year students were heavily occupied with preparing for postgraduate entrance examinations or job-seeking activities, resulting in lower response rates among senior cohorts. Second-year students had relatively more flexible schedules and were more accessible on campus. Consequently, this group showed the highest participation rate. We do not view this as a major threat to the validity of our findings, as the sample still includes substantial representation from all 4 years, and age and academic year were included as covariates in all statistical analyses to control for potential grade-related differences. The study was reviewed and approved by the Psychological Counseling Center of Guangdong University of Finance and Economics (Approval No. GDUFE-PSY-2026-021).

### 2.2 Measures

#### 2.2.1 Involution perception

Involution perception was assessed using the revised Chinese Involution Perception Scale (Zhang et al., 2024). The scale consists of 18 items rated on a 7-point Likert scale ranging from 1 (strongly disagree) to 7 (strongly agree), with higher mean scores indicating stronger perceived pressure from a competitive environment. The scale encompasses four theoretically derived dimensions: Psychological Pressure (e.g.,” I feel mentally exhausted by the constant need to outperform others“), Social Norms (e.g.,” In my environment, overwork is seen as a virtue rather than a problem“), Competitive Behaviors (e.g.,” My peers routinely engage in activities solely to enhance their resumes“), and Resource Scarcity (e.g.,” Opportunities such as scholarships and internships seem to be shrinking“). In the present sample, Cronbach’s alpha for the full scale was 0.762. The four subscales yielded Cronbach’s alphas of 0.78 (Psychological Pressure), 0.75 (Social Norms), 0.71 (Competitive Behaviors), and 0.74 (Resource Scarcity), indicating acceptable internal consistency for each dimension. Scale scores were calculated as mean item scores across all 18 items, with higher values reflecting greater involution perception. Consistent with the original validation study (Zhang et al., 2024), we used the total mean score as a global indicator of perceived involution, as the four dimensions are conceptually linked and were shown to load onto a higher-order construct, supporting the use of a composite score.

Confirmatory factor analysis (CFA) supported the four-factor structure of the scale in the present sample with acceptable fit: *χ*^2^(129) = 586.32, CFI = 0.961, TLI = 0.955, RMSEA = 0.059, 90% CI [0.054, 0.064], SRMR = 0.043. All standardized factor loadings were statistically significant and ranged from 0.52 to 0.78. Composite reliability values for the four subscales ranged from 0.71 to 0.82.

#### 2.2.2 Problematic smartphone use

PSU was assessed using the Smartphone Addiction Scale–Short Version (SAS-SV; Kwon et al., 2013). The SAS-SV contains 10 items rated on a 6-point Likert scale ranging from 1 (strongly disagree) to 6 (strongly agree), with higher mean scores indicating more severe problematic smartphone use. A sample item is: “I have a hard time concentrating in class, while doing assignments, or while working due to smartphone use.” In the present sample, Cronbach’s alpha was 0.899. CFA results indicated acceptable fit: *χ*^2^(35) = 232.07, CFI = 0.952, TLI = 0.939, RMSEA = 0.075, 90% CI [0.068, 0.082], SRMR = 0.047. Standardized factor loadings ranged from 0.61 to 0.82.

#### 2.2.3 Anxiety

Anxiety was measured using the Generalized Anxiety Disorder-7 scale (GAD-7; Spitzer et al., 2006). The GAD-7 consists of seven items assessing the frequency of anxiety symptoms over the past 2 weeks on a 4-point Likert scale ranging from 0 (not at all) to 3 (nearly every day). A sample item is: “Feeling nervous, anxious, or on edge.” Higher mean scores indicate greater anxiety severity. In the present sample, Cronbach’s alpha was 0.923. CFA results indicated acceptable fit: *χ*^2^(14) = 82.67, CFI = 0.980, TLI = 0.970, RMSEA = 0.070, 90% CI [0.059, 0.081], SRMR = 0.028. Standardized factor loadings ranged from 0.71 to 0.87.

#### 2.2.4 Psychological resilience

Psychological resilience was measured using the 10-item Connor-Davidson Resilience Scale (CD-RISC-10; Campbell-Sills and Stein, 2007). The Chinese psychometric properties of the CD-RISC-10 have been validated in Chinese college samples (Chen et al., 2021). The CD-RISC-10 assesses the ability to cope with adversity and stress over the past month on a 5-point Likert scale ranging from 0 (not true at all) to 4 (true nearly all the time). A sample item is: “I am able to adapt when changes occur.” Higher mean scores indicate greater resilience. In the present sample, Cronbach’s alpha was 0.927. CFA results indicated acceptable fit: *χ*^2^(35) = 183.02, CFI = 0.966, TLI = 0.956, RMSEA = 0.065, 90% CI [0.058, 0.072], SRMR = 0.036. Standardized factor loadings ranged from 0.65 to 0.84.

#### 2.2.5 Control variables

Age (in years) and gender (0 = female, 1 = male) were included as covariates in all analyses to account for potential demographic influences on the variables of interest.

### 2.3 Common method bias assessment

To address concerns regarding common method variance inherent to cross-sectional self-report designs, several procedural and statistical remedies were employed. Procedurally, the survey was administered anonymously, and participants were assured that there were no right or wrong answers to reduce evaluation apprehension. Items from different scales were interspersed to minimize consistency motives. Statistically, Harman’s single-factor test indicated that the first unrotated factor accounted for 25.65% of the total variance, which fell substantially below the conventional 50% threshold. Although this result suggests that common method bias was not a dominant concern, it cannot definitively rule out shared method variance. Additionally, the multi-factor CFA model in which items loaded onto their respective latent constructs demonstrated significantly better fit than a single-factor model: Δ*χ*^2^(6) = 2,847.53, *p* < 0.001, providing further evidence for the discriminant validity of the measures.

### 2.4 Data analysis plan

Descriptive statistics, internal consistency coefficients, and Pearson correlations were computed using SPSS 29.0. The hypothesized moderated mediation model was tested using the PROCESS macro for SPSS (version 3.3; Hayes, 2022). Specifically, Model 8 was specified, which estimates the moderating effect of psychological resilience on both the a path (involution perception → anxiety) and the c′ path (the direct effect of involution perception on PSU), thereby allowing for a conditional indirect effect.

All continuous predictor and moderator variables were mean-centered prior to the construction of the interaction term to reduce multicollinearity and facilitate the interpretation of conditional effects at meaningful values of the moderator. The indirect effect was quantified as the product of the a path and the b path (anxiety → PSU) at low (*M* − 1 SD), mean (*M*), and high (*M* + 1 SD) levels of psychological resilience. Bias-corrected 95% confidence intervals were generated using 5,000 bootstrap resamples. An indirect effect was considered statistically significant if the 95% confidence interval did not include zero. The index of moderated mediation was also computed to test whether the indirect effect varied linearly as a function of psychological resilience. Age and gender were entered as covariates in both equations.

## 3 Results

### 3.1 Preliminary analyses

All continuous predictor and moderator variables were mean-centered prior to the main analyses to reduce multicollinearity and facilitate interpretation. Thus, in the moderated mediation model, the conditional effects of involution perception at low, mean, and high levels of psychological resilience represent values at one standard deviation below the mean, at the mean (i.e., zero after centering), and one standard deviation above the mean, respectively.

Descriptive statistics and bivariate correlations for all study variables are presented in Table 1. Involution perception was positively correlated with PSU (*r* = 0.32, *p* < 0.001) and anxiety (*r* = 0.39, *p* < 0.001). PSU was also positively correlated with anxiety (*r* = 0.38, *p* < 0.001). Psychological resilience was negatively correlated with involution perception (*r* = −0.21, *p* < 0.001), PSU (*r* = −0.40, *p* < 0.001), and anxiety (*r* = −0.44, *p* < 0.001). The mean item scores (based on raw, uncentered data) were 4.07 (SD = 0.56) for involution perception, 3.18 (SD = 0.93) for PSU, 1.77 (SD = 0.61) for anxiety, and 3.65 (SD = 0.63) for psychological resilience. Internal consistency was acceptable to excellent across all measures. Age was significantly positively correlated with PSU (*r* = 0.09, *p* = 0.005), and gender was significantly correlated with psychological resilience (*r* = 0.12, *p* < 0.001) and anxiety (*r* = −0.08, *p* = 0.009).

**Table 1:** Descriptive statistics, internal consistency, and bivariate correlations among study variables (*N* = 1,002).

Variable	*M*	SD	*α*	1	2	3	4
1. Involution perception	4.07	0.56	0.762	—			
2. Problematic smartphone use	3.18	0.93	0.899	0.32***	—		
3. Anxiety	1.77	0.61	0.923	0.39***	0.38***	—	
4. Psychological resilience	3.65	0.63	0.927	−0.21***	−0.40***	−0.44***	—

M, mean item score (raw, uncentered); SD, standard deviation; *α*, Cronbach’s alpha. All scales were calculated as mean item scores. ***p < 0.001.

### 3.2 Confirmatory factor analyses

Confirmatory factor analyses were conducted to examine the factor structure of each measure. As shown in Table 2, all scales demonstrated acceptable to good fit based on conventional reference criteria (Hu and Bentler, 1999): CFI ≥ 0.90 acceptable, ≥ 0.95 good; TLI ≥ 0.90 acceptable, ≥ 0.95 good; RMSEA ≤ 0.08 acceptable, ≤ 0.06 good; SRMR ≤ 0.08 acceptable. The four-factor model of the Involution Perception Scale showed good fit; the single-factor models of the PSU, Anxiety, and Psychological Resilience scales also showed acceptable to good fit. All standardized factor loadings were statistically significant (*p* < 0.001) and ranged from 0.52 to 0.87 across scales, supporting the construct validity of the measures.

**Table 2:** Confirmatory factor analysis fit indices for all study measures.

Scale	*χ* ^2^	df	CFI	TLI	RMSEA	RMSEA 90% CI	SRMR	Fit evaluation
Involution Perception (4-factor)	586.32	129	0.961	0.955	0.059	[0.054, 0.064]	0.043	Good
PSU (SAS-SV) (1-factor)	232.07	35	0.952	0.939	0.075	[0.068, 0.082]	0.047	Acceptable to Good
Anxiety (GAD-7) (1-factor)	82.67	14	0.980	0.970	0.070	[0.059, 0.081]	0.028	Good
Psychological Resilience (CD-RISC-10) (1-factor)	183.02	35	0.966	0.956	0.065	[0.058, 0.072]	0.036	Good

CFI, Comparative Fit Index; TLI, Tucker–Lewis Index; RMSEA, Root Mean Square Error of Approximation; SRMR, Standardized Root Mean Square Residual. Reference criteria: CFI ≥ 0.90 acceptable, ≥ 0.95 good; TLI ≥ 0.90 acceptable, ≥ 0.95 good; RMSEA ≤ 0.08 acceptable, ≤ 0.06 good; SRMR ≤ 0.08 acceptable (Hu and Bentler, 1999). All factor loadings were statistically significant (*p < 0.001).

### 3.3 Moderated mediation model

**Figure 1:**
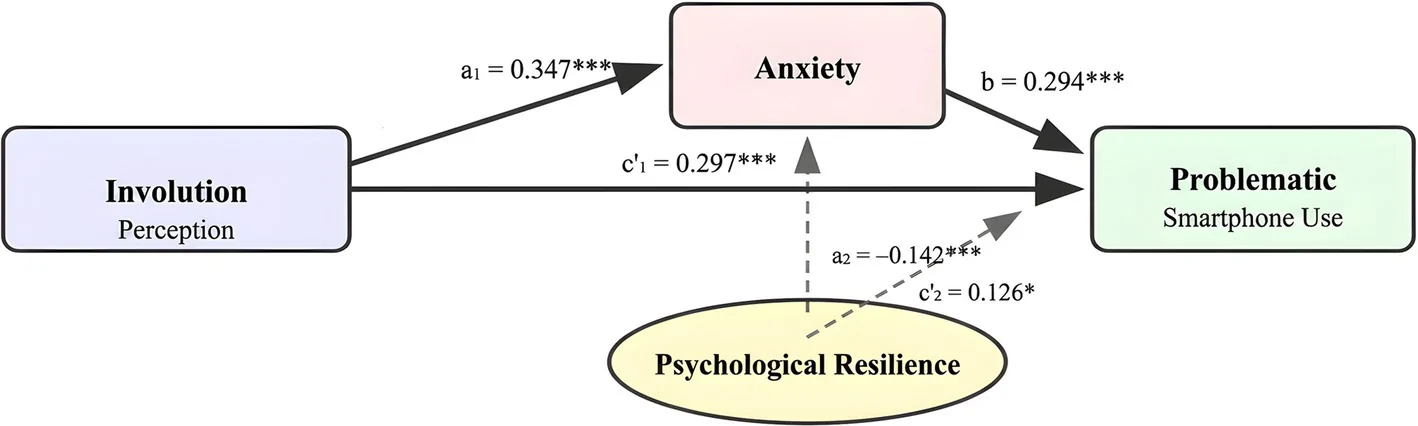
Conceptual moderated mediation model with unstandardized path coefficients. *p < 0.05, ***p < 0.001. Age and gender were included as covariates in all equations. Colors are used to distinguish variable roles for visual clarity. All figures were created using R (version 4.3.1) with the ‘ggplot2’ and ‘ggpubr’ packages.

The moderated mediation model (PROCESS Model 8) was tested with mean-centered continuous variables. Unstandardized regression coefficients are presented in Table 3. The conceptual model with path coefficients is depicted in Figure 1.

**Table 3:** Moderated mediation model results: Unstandardized regression coefficients from PROCESS Model 8.

Predictor	*b*	*SE*	*t*	*p*	95% CI
Outcome: anxiety (*R*^2^ = 0.29, *F*(5, 996) = 81.37, *p* < 0.001)					
Involution perception	0.347	0.031	11.19	< 0.001	[0.286, 0.408]
Psychological resilience	−0.295	0.028	−10.54	< 0.001	[−0.350, −0.240]
Involution × resilience	−0.142	0.034	−4.20	< 0.001	[−0.209, −0.076]
Outcome: problematic smartphone use (*R*^2^ = 0.25, *F*(5, 996) = 66.40, *p* < 0.001)					
Involution perception	0.297	0.044	6.75	< 0.001	[0.211, 0.383]
Anxiety	0.294	0.040	7.43	< 0.001	[0.216, 0.372]
Psychological resilience	−0.297	0.043	−6.91	< 0.001	[−0.381, −0.213]
Involution × resilience	0.126	0.053	2.38	0.017	[0.022, 0.230]

*N* = 1,002. All continuous predictor and moderator variables were mean-centered. *b*, unstandardized coefficient; SE, standard error; CI, confidence interval. Age and gender were controlled in both equations (coefficients not displayed for parsimony).

#### 3.3.1 Anxiety equation

As shown in the upper panel of Table 3, involution perception was positively associated with anxiety, *b* = 0.347, SE = 0.031, *t* = 11.19, *p* < 0.001. The main effect of psychological resilience on anxiety was negative and significant, *b* = −0.295, SE = 0.028, *t* = −10.54, *p* < 0.001. Critically, the interaction between involution perception and psychological resilience was significant, *b* = −0.142, SE = 0.034, *t* = −4.20, *p* < 0.001, indicating that psychological resilience attenuated the positive association between involution perception and anxiety. The model accounted for 29% of the variance in anxiety, *R*^2^ = 0.29, *F*(5, 996) = 81.37, *p* < 0.001.

#### 3.3.2 PSU equation

As shown in the lower panel of Table 3, anxiety was positively associated with PSU, *b* = 0.294, SE = 0.040, *t* = 7.43, *p* < 0.001. Psychological resilience was negatively associated with PSU, *b* = −0.297, SE = 0.043, *t* = −6.91, *p* < 0.001. The direct effect of involution perception on PSU (after controlling for anxiety and the interaction term) was also significant, *b* = 0.297, SE = 0.044, *t* = 6.75, *p* < 0.001. Importantly, the interaction between involution perception and psychological resilience on PSU was significant, *b* = 0.126, SE = 0.053, *t* = 2.38, *p* = 0.017. The positive coefficient indicates that the direct association between involution perception and PSU became stronger, rather than weaker, at higher levels of psychological resilience. The model accounted for 25% of the variance in PSU, *R*^2^ = 0.25, *F*(5, 996) = 66.40, *p* < 0.001.

### 3.4 Conditional indirect and direct effects

As shown in Table 4, the indirect association between involution perception and PSU through anxiety was significant at all three levels of psychological resilience. Specifically, the indirect effect was strongest at low resilience [effect = 0.128, Boot SE = 0.023, 95% CI (0.085, 0.173)], followed by mean resilience [effect = 0.102, Boot SE = 0.017, 95% CI (0.069, 0.135)] and high resilience [effect = 0.075, Boot SE = 0.015, 95% CI (0.046, 0.104)]. Because none of the confidence intervals included zero, the indirect association was statistically significant at all levels of resilience. Supporting this pattern, the index of moderated mediation was negative and significant, index = −0.042, Boot SE = 0.011, 95% CI [−0.064, −0.020], indicating that the strength of the indirect effect decreased linearly as psychological resilience increased.

**Table 4:** Conditional indirect and direct effects of involution perception on PSU at low, mean, and high levels of psychological resilience.

Effect Type	Level of resilience	Effect	Boot *SE*	95% CI lower	95% CI upper
Indirect effect (via anxiety)	Low (*M* − 1 *SD*)	0.128	0.023	0.085	0.173
Indirect effect (via anxiety)	Mean (*M*)	0.102	0.017	0.069	0.135
Indirect effect (via anxiety)	High (*M* + 1 *SD*)	0.075	0.015	0.046	0.104
Direct effect	Low (*M* − 1 *SD*)	0.217	0.058	0.104	0.331
Direct effect	Mean (*M*)	0.297	0.043	0.213	0.381
Direct effect	High (*M* + 1 *SD*)	0.377	0.053	0.273	0.481

*N* = 1,002. Bootstrap estimates based on 5,000 resamples. CI, confidence interval. All effects are unstandardized. Low = *M* − 1 SD; Mean, *M*; High = *M* + 1 SD of psychological resilience. Age and gender were controlled in all analyses. Index of moderated mediation = −0.042, Boot SE = 0.011, 95% CI [−0.064, −0.020].

The conditional direct association between involution perception and PSU was also significant at all three levels. In contrast to the indirect effect, the direct effect was weakest at low resilience [effect = 0.217, Boot SE = 0.058, 95% CI (0.104, 0.331)], followed by mean resilience [effect = 0.297, Boot SE = 0.043, 95% CI (0.213, 0.381)], and strongest at high resilience [effect = 0.377, Boot SE = 0.053, 95% CI (0.273, 0.481)]. Thus, the direct positive association between involution perception and PSU became progressively stronger as psychological resilience increased.

### 3.5 Simple slope analyses

**Figure 2:**
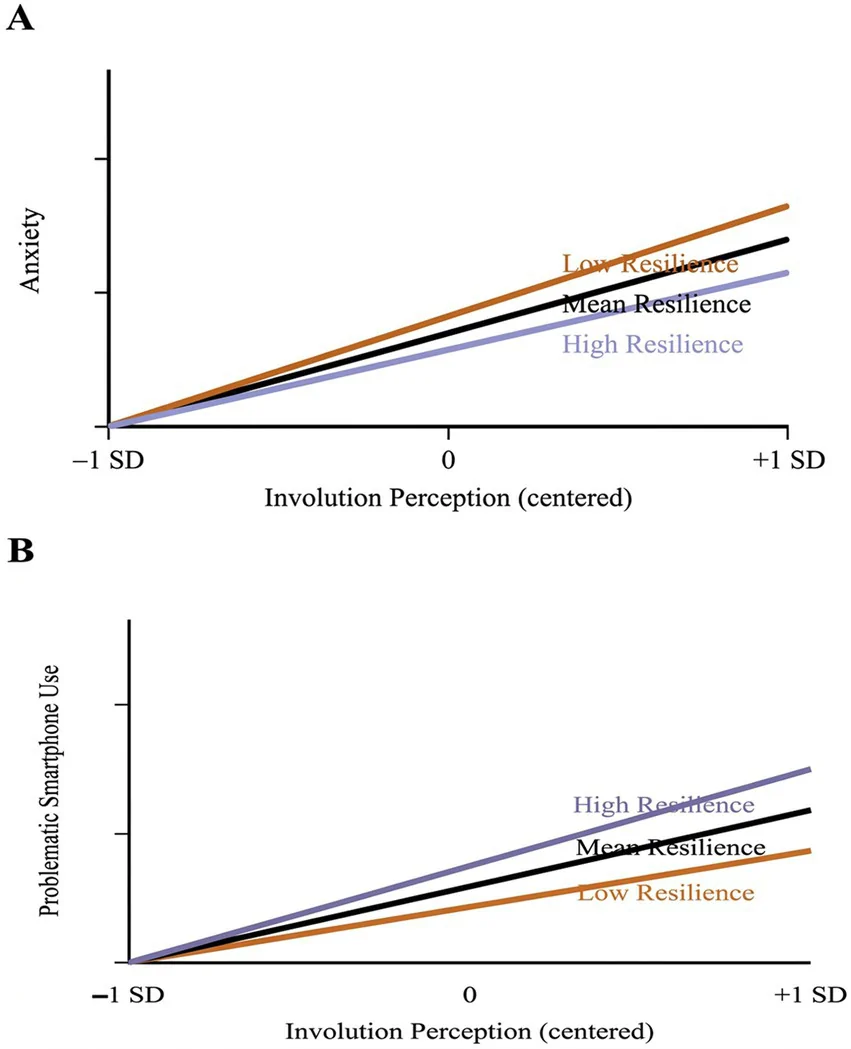
Simple slope analyses: Interaction effects at low, mean, and high levels of psychological resilience. Slopes are unstandardized simple slopes from PROCESS Model 8. All slopes significant at *p* < 0.001. Low = *M* − 1 *SD*; Mean = *M*; High = *M* + 1 *SD* of psychological resilience. **(A)** Anxiety as outcome. **(B)** Problematic smartphone use as outcome.

The simple slope analyses are displayed in Figure 2. For anxiety (Panel A), the slope of involution perception on anxiety was 0.436 at low resilience, 0.347 at mean resilience, and 0.257 at high resilience. All three simple slopes were significant (*p* < 0.001). The positive association between involution perception and anxiety became progressively weaker as psychological resilience increased (simple slopes: low resilience = 0.436, mean resilience = 0.347, and high resilience = 0.257, showing a progressively decreasing pattern), supporting H3.

For PSU (Panel B), the direct slope of involution perception on PSU was 0.217 at low resilience (*p* < 0.001), 0.297 at mean resilience (*p* < 0.001), and 0.377 at high resilience (*p* < 0.001). In contrast to the pattern observed in Panel A, the direct association between involution perception and PSU became progressively stronger as psychological resilience increased (simple slopes: low resilience = 0.217, mean resilience = 0.297, and high resilience = 0.377, showing a progressively increasing pattern). This pattern is consistent with the sustained engagement perspective rather than the global protective model.

The key observation from Figure 2 is the opposite ordering of the simple slopes across the two panels: psychological resilience attenuated the slope of the emotional pathway (Panel A) while amplifying the slope of the direct behavioral pathway (Panel B). This opposite slope ordering reflects the pathway-specific moderating role of resilience, indicating that resilience does not operate uniformly across all pathways.

### 3.6 Summary of hypothesis testing

H1, which predicted a positive association between involution perception and PSU, was supported at both the correlational level (Table 1) and in the conditional direct effects (Table 4). H2, which predicted that anxiety would mediate this association, was supported by significant indirect effects at all three levels of resilience and a significant index of moderated mediation. H3, which predicted that resilience would attenuate the involution–anxiety association, was supported by the significant negative interaction in the anxiety equation (Table 3; *b* = −0.142, *t* = −4.20, *p* < 0.001). H4, which explored whether resilience moderated the direct involution–PSU association, revealed a significant positive interaction (Table 3; *b* = 0.126, *t* = 2.38, *p* = 0.017), indicating that this direct pathway was amplified rather than attenuated at higher resilience levels.

## 4 Discussion

The present study examined whether involution perception was associated with problematic smartphone use (PSU) among Chinese college students, whether anxiety statistically mediated this association, and whether psychological resilience moderated both the involution–anxiety and involution–PSU pathways. By integrating the I-PACE model and COR theory, the findings revealed a nuanced pattern: anxiety mediated the association between involution perception and PSU, while psychological resilience exhibited pathway-specific moderating roles. Specifically, resilience buffered the emotional pathway (attenuating the involution–anxiety link) yet amplified the direct behavioral pathway (strengthening the involution–PSU link). This pattern challenges the assumption that resilience is a uniformly protective factor and instead suggests a more complex, context-dependent role.

Supporting H1, involution perception was positively associated with PSU at both the bivariate and conditional-effect levels. Students who more strongly perceived their environment as characterized by intense competitive pressure, internalized overwork norms, and scarce opportunities were more likely to report maladaptive smartphone use patterns. This finding aligns with the I-PACE model’s emphasis on distal contextual factors as triggers for problematic technology use (Brand et al., 2016, 2019). Involution perception may function as a chronic, low-control stressor in which effort investment yields diminishing returns, which statistical models show to be associated with coping responses that offer immediate and accessible relief. Smartphones, as ubiquitous devices blending hedonic and instrumental functions, are ideally positioned to serve this role.

COR theory (Hobfoll, 1989) further illuminates this process. The four dimensions of involution perception may differentially contribute to resource threat: psychological pressure directly taxes emotional reserves, social norms glorifying overwork impose external demands that feel obligatory, competitive behaviors modeled by peers signal that disengagement risks falling behind, and resource scarcity perceptions narrow the perceived opportunity horizon, intensifying the sense that current effort is insufficient. Together, these dimensions may contribute to a pattern consistent with a resource loss spiral, wherein smartphones appear to be used as a readily available means of temporary replenishment. This replenishment may occur through distraction, social connection, or information seeking. These behaviors may persist despite the potential long-term costs of smartphone use to academic functioning and wellbeing.

The mediating role of anxiety was also supported by the data. Supporting H2, anxiety significantly mediated the involution–PSU association. This indirect effect remained significant across all levels of psychological resilience, although it weakened as resilience increased (Table 4: Low = 0.128, Mean = 0.102, High = 0.075). The finding is consistent with extensive evidence linking emotional distress, particularly anxiety, to problematic smartphone use (Canale et al., 2021; Elhai, 2019; Elhai et al., 2021). In the context of perceived involution, anxiety may arise from heightened vigilance regarding academic standing, future employability, and social comparison. The competitive behaviors dimension may be especially anxiogenic, as observing peers’ relentless self-optimization fosters anticipatory anxiety about one’s relative position. Smartphones may then be used for multiple anxiety-regulating functions: distraction from ruminative worry, reassurance seeking via social media, or momentary escape through entertainment. Importantly, because anxiety was assessed as a general tendency rather than context-specific academic anxiety, the observed indirect effect likely reflects a broad affective vulnerability rather than a narrowly circumscribed academic worry. Future research incorporating domain-specific anxiety measures may clarify whether academic anxiety, social anxiety, or generalized anxiety differentially accounts for the involution–PSU link.

A central contribution of this study lies in the divergent moderating effects of psychological resilience across the emotional and behavioral pathways. Consistent with H3 and the traditional resilience literature, the involution–anxiety association was significantly weaker at higher levels of resilience (simple slopes: Low = 0.436, Mean = 0.347, High = 0.257; all *p* < 0.001; Figure 2, Panel A). Students with greater capacity to adapt to adversity may possess more effective emotion regulation strategies, more flexible cognitive appraisals, or stronger social support networks, which may help account for the weaker association between competitive pressure and heightened anxiety observed among these students (Connor and Davidson, 2003; Southwick et al., 2014). Within the COR framework, resilience may function as a buffering personal resource that reduces the emotional cost of perceived resource threats.

However, the exploration of H4 revealed an unexpected pattern from a global protective standpoint: the direct association between involution perception and PSU became stronger, not weaker, at higher levels of resilience (simple slopes: Low = 0.217, Mean = 0.297, High = 0.377; all *p* < 0.001; Figure 2, Panel B). This pattern is the opposite of that observed in Panel A, reflecting the pathway-specific nature of resilience’s moderating role. Several complementary explanations may account for this finding. First, from a sustained engagement perspective, resilience may facilitate continued goal pursuit and active coping in demanding environments (Britt et al., 2016). Highly resilient students in competitive settings may remain more behaviorally immersed in academic and social ecosystems where smartphone use is functionally embedded—for scheduling, group coordination, accessing learning platforms, and maintaining professional networks. Under chronic involution pressure, this intensive, instrumentally motivated use may blur into patterns that resemble PSU. Second, the measurement overlap hypothesis suggests that the SAS-SV may not fully differentiate between excessive but functional use and truly dysregulated, addictive use. Resilient students may endorse items related to time spent or perceived dependence not because they are psychologically dependent but because their high-effort lifestyle necessitates constant connectivity. Third, from a resource investment perspective, resilient individuals may perceive smartphones as legitimate tools for preserving their competitive edge, thereby increasing device reliance. Because the present study did not assess specific smartphone-use functions, all three interpretations remain tentative and await direct empirical examination.

This pathway-specific pattern suggests that resilience should not be operationalized as a monolithic protective trait in models of maladaptive technology use. Instead, its effects may depend on the specific pathway under examination, echoing recent critiques that resilience, particularly in high-demand environments, may promote persistence to the point of overextension (Britt et al., 2016).

The present findings carry several implications for university mental health practice. First, given the robust association between involution perception and PSU, interventions should address not only individual smartphone habits but also the broader perceived competitive environment. University-level initiatives that promote cooperative learning, redefine success beyond relative rankings, and expand perceived opportunity structures may reduce the zero-sum mindset that fuels involution perception. The social norms dimension may be amenable to normative feedback interventions that correct misperceptions about peers’ actual study habits and wellbeing.

Second, anxiety represents a modifiable intermediate target. Evidence-based interventions such as cognitive-behavioral therapy and mindfulness-based stress reduction have demonstrated efficacy in reducing both anxiety and problematic technology use. Embedding such programs within student counseling services could disrupt the indirect pathway documented here.

Third, and most critically, the pathway-specific resilience effect suggests that one-size-fits-all resilience-building programs may be insufficient if the goal is reducing PSU. While resilience training may effectively reduce emotional distress, it may not automatically mitigate behavioral dependence on smartphones in competitive environments. Interventions should therefore be two-pronged: resilience-building to manage emotional responses to competition, paired with explicit training in digital boundary management, offline coping repertoires, and awareness of when intensive functional smartphone use transitions into impairment.

Given the cross-sectional nature of the present data, all path coefficients and indirect effects should be interpreted as statistical associations rather than causal effects. The moderated mediation model tests a theoretically derived pattern of relationships, but it does not establish temporal precedence or causal direction. Longitudinal and experimental studies are needed to test the directional hypotheses implied by the I-PACE model and COR theory.

Several limitations should be considered. First, the cross-sectional design precludes causal inferences. Longitudinal designs would substantially strengthen causal claims. Such designs could include ecological momentary assessment to capture within-person fluctuations; future research should incorporate objective behavioral indicators of smartphone use and multi-informant assessments. Third, the sample was recruited from a single finance and economics university in Guangzhou, limiting generalizability. Replication in multi-site, nationally representative samples is needed. Fourth, the Cronbach’s alpha for the Involution Perception Scale (0.762) was acceptable but moderate, likely reflecting its multidimensional structure. Future psychometric work should aim to improve item homogeneity within dimensions or explore whether a bifactor model better represents the data. Fifth, PSU was treated as a unidimensional construct; different smartphone functions (e.g., social media, gaming, academic coordination) may show distinct associations with involution perception and anxiety. Sixth, unmeasured confounders such as personality traits (e.g., neuroticism, conscientiousness) and family socioeconomic status may affect the observed associations. Future studies should include a broader array of covariates.

Despite these limitations, the present study extends the nomological network of involution, provides a theoretically coherent account of how culturally specific competitive pressures relate to PSU, and identifies a pathway-specific moderating role of psychological resilience that challenges deficit-focused models of PSU.

## 5 Conclusion

The present study examined the associations among involution perception, anxiety, psychological resilience, and PSU in a sample of Chinese undergraduate students. Three principal findings emerged. First, involution perception encompasses the subjective experience of psychological pressure, internalized social norms of overwork, perceived competitive behaviors among peers, and a sense of resource scarcity. This perception was positively associated with PSU both directly and indirectly through heightened anxiety. This finding extends the I-PACE model and COR theory by suggesting that culturally embedded competitive pressures represent meaningful distal correlates of maladaptive technology use in higher education settings.

Second, anxiety statistically mediated the involution–PSU association, and this indirect pathway was moderated by psychological resilience. Consistent with the traditional protective view, higher resilience was associated with a weaker emotional response to involution, such that the involution–anxiety association was weaker among highly resilient students (simple slope = 0.257 vs. 0.436 at low resilience). Consequently, the anxiety-mediated pathway to PSU was reduced among these students (indirect effect = 0.075 vs. 0.128 at low resilience).

Third, psychological resilience demonstrated a pathway-specific moderating role on the direct behavioral pathway. Rather than buffering against PSU, higher resilience amplified the direct association between involution perception and PSU (simple slope = 0.377 vs. 0.217 at low resilience). This finding is the opposite of the pattern observed in the anxiety pathway and suggests that resilience does not function as a uniformly protective factor in competitive academic environments. While resilience may safeguard emotional wellbeing, it may simultaneously be associated with sustained, high-intensity engagement with smartphones. This pattern may reflect a process in which adaptive persistence blurs into excessive behavioral reliance. Because smartphone-use functions were not assessed in this study, this interpretation remains tentative.

Taken together, these findings carry both theoretical and practical implications. Theoretically, they underscore the need to conceptualize resilience as a pathway-dependent moderator whose effects may diverge across emotional and behavioral outcomes. Practically, they suggest that interventions aimed at reducing PSU in competitive educational contexts should adopt a dual-pronged approach: strengthening emotional coping resources to mitigate anxiety while simultaneously cultivating awareness of when high-frequency functional smartphone use transitions into impairment. University mental health initiatives that address involution perception directly may complement individual-level interventions in reducing the psychological burden associated with maladaptive smartphone use. These initiatives could do so by challenging distorted social norms, expanding perceived opportunity structures, and fostering cooperative rather than zero-sum definitions of success.

Future longitudinal, multi-method, and multi-site studies are needed to replicate these findings, clarify the temporal dynamics among involution perception, anxiety, and PSU, and disentangle the specific functions of smartphone use that underlie the pathway-specific resilience effect.

## Data Availability

The raw data supporting the conclusions of this article will be made available by the authors, without undue reservation.
